# Using Sleep Time Data from Wearable Sensors for Early Detection of Migraine Attacks

**DOI:** 10.3390/s18051374

**Published:** 2018-04-28

**Authors:** Pekka Siirtola, Heli Koskimäki, Henna Mönttinen, Juha Röning

**Affiliations:** Biomimetics and Intelligent Systems Group, University of Oulu, P.O. BOX 4500, Oulu FI-90014, Finland; heli.koskimaki@oulu.fi (H.K.); henna.monttinen@oulu.fi (H.M.); juha.roning@oulu.fi (J.R.)

**Keywords:** migraine, early detection, wearable sensors, machine learning

## Abstract

The migraine is a chronic, incapacitating neurovascular disorder, characterized by attacks of severe headache and autonomic nervous system dysfunction. Among the working age population, the costs of migraine are 111 billion euros in Europe alone. The early detection of migraine attacks would reduce these costs, as it would shorten the migraine attack by enabling correct timing when taking preventive medication. In this article, whether it is possible to detect migraine attacks beforehand using wearable sensors is studied, and t preliminary results about how accurate the recognition can be are provided. The data for the study were collected from seven study subjects using a wrist-worn Empatica E4 sensor, which measures acceleration, galvanic skin response, blood volume pulse, heart rate and heart rate variability, and temperature. Only sleep time data were used in this study. A novel method to increase the number of training samples is introduced, and the results show that, using personal recognition models and quadratic discriminant analysis as a classifier, balanced accuracy for detecting attacks one night prior is over 84%. While this detection rate is high, the results also show that balance accuracy varies greatly between study subjects, which shows how complicated the problem actually is. However, at this point, the results are preliminary as the data set contains only seven study subjects, so these do not cover all migraine types. If the findings of this article can be confirmed in a larger population, it may potentially contribute to early diagnosis of migraine attacks.

## 1. Introduction

The migraine is a neurological disorder, usually associated with attacks of severe headache, nausea, vomiting, or sensitivity to light, sound, or movement and, when untreated, typically lasts 4–72 h ([1,2,3]). Moreover, the migraine is the number one cause of disability that occurs in under 50 s [4]. Due to its commonness, 15% of people in European countries suffer from migraines [5], it is also an expensive disease. The costs of migraine are 111 billion euros among the working age population in Europe alone [6]. These are mainly caused by lost working days.

A migraine attack can consist of five phases: prodrome (e.g., food craving), aura (e.g., visual, sensory, or motor symptoms preceding the headache), headache (usually unilateral, pulsating), resolution (pain wanes), and recovery [7]. However, the duration and existence of these phases differ among individuals. Moreover, there is a great amount of types of migraine attacks, making each one highly particular. For example, according to the International Headache Society [8], migraine types can be divided into five main categories (migraines without auras, migraines with auras, migraines with brainstem auras, hemiplegic migraines, and reginal migraines), and these can be further divided into subcategories.

The medication of migraine can be divided into two categories: preventive (daily dose) and acute (when symptoms start). Preventive medication is expensive and is avoided for as long as possible [9]. On the other hand, the problem with acute medication is that some people do not have early symptoms. In most cases, people tend to dismiss early symptoms. Thus, the early detection of migraine attacks would be a valuable addition to treatment for those using only acute medication.

In this article, preliminary results of the early detection of migraine attacks based on human biosignals collected with wearable sensors are presented. In our approach, the idea is to use a single, easy, and comfortable wrist-worn device to measure 24/7 data from study subjects to develop personal models for early migraine attack detection based on sleep time data. While the sleep time recognition is not an optimal solution for acute medication purposes, the user can be informed to become more alert for their early symptoms based on the fact that 87.8% of people having migraines (*N* = 565) are also recognized as having pre-symptoms [10]. On the other hand, the sleep time recognition can also be considered as a preliminary step needed to build a more accurate prediction system, where only days on which a migraine is going to occur are screened in more detail, e.g., by increasing additional sensors, employing new methodologies, or taking extra cognitive measurements during the day. Moreover, in this article, the number of data points is increased using a novel approach. Using this approach and machine learning models, the aim of this study is to give answers to the following research questions:

1. Is it possible to recognize a migraine attack beforehand using wearable sensors?
2. Should the recognition be based on personal or user-independent prediction models?

The paper is organized as follows: Related work is introduced in Section 2, and Section 3 introduces the used data collection and a method to increase the number of observations. Section 4 explains how sleep time data were used in the recognition process. The results of the experiments are shown in Section 5. Finally, the discussion is in Section 6 and conclusions are in Section 7.

## 2. Related Work

The aim of this study is to build reliable recognition models for the early detection of migraine attacks based on sleep time data collected using wearable sensors. The study is based on two hypotheses: (1) wearable sensor data can be used to detect an attack of illness beforehand, and (2) sleep time data contains information about the forthcoming migraine attack. Related work supports both of these hypotheses. Firstly, it has been shown that wearable sensor data can be used to analyze sleep; for instance, in [11,12], wearable sensors have been used to detect sleeping patterns and stages. Moreover, there is a known relationship between sleep disturbances and migraines, and this association is especially strong between nighttime and early morning migraine attacks, and sleep problems [13]. Secondly, there are studies showing that wearable sensor data can be used for an early diagnosis at least in some diseases and seizures. For instance, in [14], upcoming epileptic seizures were predicted based on heart rate variability, which can be measured optically using wearable sensors. In epilepsy, the pre-ictal period affects the autonomic nervous systems, which affects heart rate variability. Therefore, the recognition was based on anomaly monitoring using multivariate statistical process control. The data set used in the study was small, but the results were promising: the data set contained 11 seizures and using the methods presented in the paper, 10 of these were recognized beforehand.

As such, early detection of migraine attacks is not a new idea. For instance, in [15], early detection was based on contingent negative variation (CNV), which is one of the neurophysiological factors that measures cortical potential and can be calculated from EEG (electroencephalography) signals. The study shows that the amplitude of CNV raises a day before a migraine attack. A different EEG-based approach was presented in [16] where migraine phases and EEG-data related to them was categorized as inter-ictal, pre-ictal, ictal, and post-ictal. The aim was to build a user-independent model to detect a migraine phase. Therefore, the approach could be used for early diagnosis. The data were collected from 108 migraine patients and classification was based on a fuzzy neural network, which managed to classify instances with an accuracy of 66%.

The studies most similar to ours are by Pagán et al. (e.g., [17,18]) where physiological signals are used to predict migraine attacks. The data for these studies are collected from only two subjects and using two devices: a finger-held SpO2 device and patches attached to the chest that can measure ECG, skin temperature, and electrodermal activity. In [17], Pagán et al. built a state-space-modeling-technique-based personalized prediction model for both study subjects and managed to warn study subjects about a forthcoming migraine attack on average 47 min before the attack. The results were even better in [18], where grammatical-evolutionary-algorithm-based recognition models were used. Using this method, it was possible to detect migraine attacks 20 min beforehand with an accuracy of around 70%. The drawback of these studies was that the data set contained data from only two subjects, so it not possible to say how well the results can be generalized and how well methods work with different migraine types. Moreover, the sensors used in these articles differ from the ones used in our study. In fact, with the technology currently in the market, the approach proposed by [17,18] cannot be implemented with a device that is supposed to be worn 24/7.

## 3. Data Set and Feature Extraction

In this section, the collected data set is introduced. In addition, the section contains information on how the data was pre-processed. Lastly, the extracted features are listed, and a novel method to increase the number of data points is introduced.

### 3.1. Collected Data Set

Data for this study were collected from seven volunteer study subjects. Physical characteristics and ages of the study subjects are presented in Table 1. Most of the study subjects were women (five women and two men), and the age of the study subjects varied from 30 to 60 years. While the subjects were different, the type of migraine they had was also different. For instance, some of them had aura symptoms, while most did not (two study subjects with aura symptoms and five without). In addition, most of them did not use any preventive medication, but all of them used medication during the migraine attacks. Common among all study subjects is that they had migraine attacks quite often. This was a criterion for joining the study, as frequent attacks enabled a shorter data collection period, which ensured that data consisted of several migraine attacks for every study subject.

The data set consists of two parts: sensor data collected using the Empatica E4 wristband [19] from Empatica Boston, USA, and a diary where study subjects described and commented their feelings and symptoms using their own words. The labeling of the sensor data was based on this diary.

Empatica E4 includes four sensors: (1) a 3D accelerometer with 32 Hz sampling frequency, (2) a thermometer with a 4 Hz sampling frequency, (3) an electrodermal activity sensor (EDA) with a 4 Hz sampling frequency, which is used to measure galvanic skin response, and (4) a photoplethysmography sensor (PPG), which can measure blood volume pulse (64 Hz), heart rate (1 Hz), and heart rate variability. As can be seen, blood volume pulse has the highest sampling rate (64 Hz), and in the pre-processing stage, all other signals were over-sampled to this same sampling frequency. This enabled calculation of the correlation between signals.

Similar instructions were given to each study subject. They were told to wear the Empatica E4 wristband 24/7 on their non-dominant hand. The reason for this is the EDA signal. The authors’ previous study [20] showed slight favor for a non-dominant hand regarding nighttime EDA asymmetry. In addition, study subjects were advised to take the device off while charging and uploading data from the device to the cloud. In addition, they were told to take off the device while showering, swimming, and going to the sauna. Altogether, 200 days’ worth of data was collected—approximately 27 days for each study subject—rendering the data set extensive (see Table 2).

### 3.2. Studying Sleep Time Data and Increasing the Number of Data Points

Although the Empatica E4 was developed to measure biosignals 24/7, in the pre-processing stage, it was noticed that the quality of signals was not good during the daytime. The reason for this was that movement seemed to cause a great deal of disturbances in the signals, especially in PPG. PPG is based on measuring the blood volume pulse optically. In order to obtain correct results, the device needs to be fitted perfectly with just enough pressure so that the LEDs and photodiodes can monitor blood flow correctly. In fact, it is explained on the Empatica website that the device cannot recognize heart rate variability while the hand is moving a great deal [21]. The reason for this is that Empatica software needs to detect several sequential heart beats to reliably measure heart rate variability, and this is not possible during physical movement. The association of physical activity and problems in heart rate variability have been studied in greater detail in [22]. Due to this very problem, it was decided to use only sleep time data in this study. Timestamps for falling asleep and waking up were estimated based on accelerometer data using visual inspection by visually searching sequences where there is noticeably less movement in 3D acceleration signals than there is during the daytime as well as sequences where such movement starts again. This procedure does not give exact timestamps for falling asleep and waking up but good approximations. In fact, exact timestamps are not needed in this study, as features are extracted from time windows that can be several hours long, so small differences between approximate and exact timestamps do not have a significant effect on feature values. Therefore, the hypothesis of this study is that, by concentrating on the signals collected during the sleep time, a rough estimate can be given about the probability of a migraine attack the next day.

To test the hypothesis, sleep data were divided into two classes: (1) nights before a day without a migraine and (2) nights before a day with a migraine. Therefore, class (2) contains information about the pre-ictal stage of a migraine attack. The idea of this approach is to tell the user when he/she wakes up in the morning if he/she is going to have a migraine attack that day and take medication if needed.

However, the problem of this approach is that, though the data set is extensive (on average 27 days per study subject), considering one night as one observation compresses the data set so much that reliable user-dependent models cannot be built based on it. What is especially problematic with this approach is that, with many study subjects, there are only data on a few nights before a day with a migraine attack, which means the data set is highly imbalanced. Therefore, there are not enough observations for meaningful cross-validation.

In order to increase the number of observations, it was decided not to train the prediction models using data extracted from each separate night. Instead, it was decided to use the differences between nights as features. Differences were calculated so that (1) nights before a migraine attack were compared to nights before a day without a migraine, and (2) nights before a day without a migraine were compared with each other.

**Figure 1:**
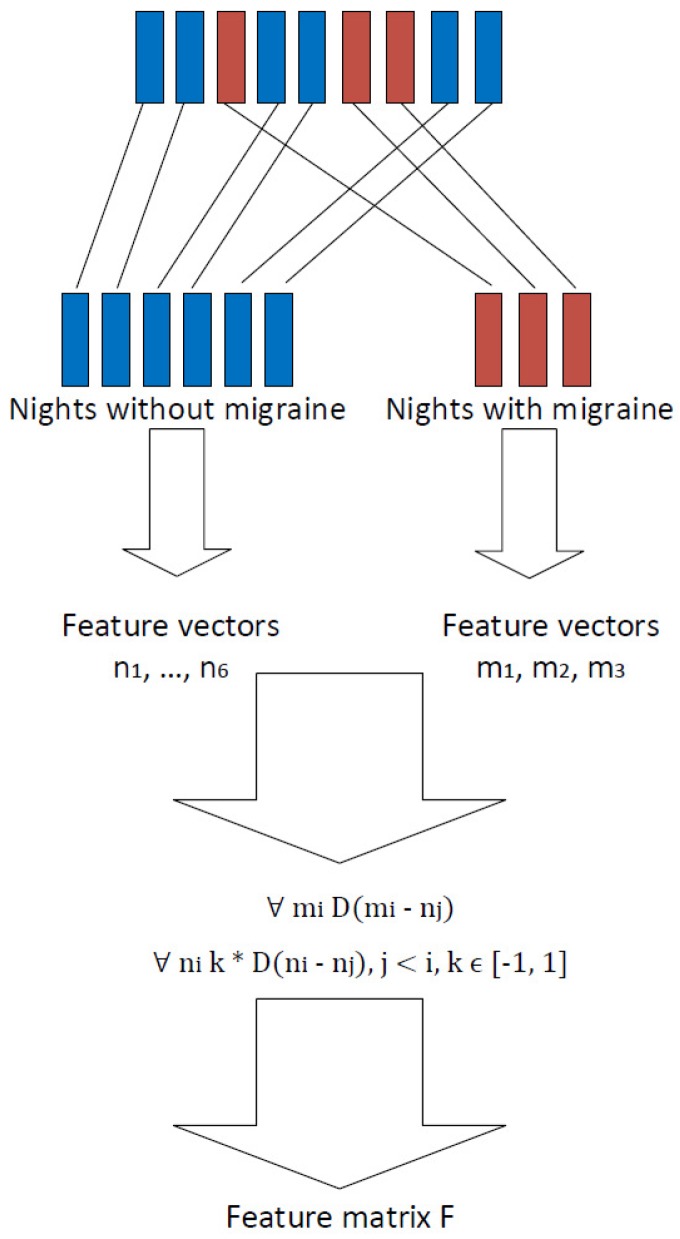
Extracting features from sleep time data.

The approach to increase the number of observations is presented in Algorithm 1 and Figure 1. As an example, let us mark $n_{k}$ as the feature vector extracted from the *k*th night before a day without a migraine attack and $m_{l}$ as the feature vector extracted from the *l*th night before a day with a migraine attack. The final feature matrix describing the differences between nights was calculated using Algorithm 1. The algorithm takes features vectors $n_{1},\ldots ,n_{a},m_{1},\ldots ,m_{b}$ from all nights as an input. Then, in the first place, nights before a migraine day $m_{i}$ are compared to nights before a non-migraine day $n_{j}$. The remainder $m_{i}-n_{j}$ is then used as a feature in the classification process, and this observation is labeled as a “migraine day.” Nights before a day without migraine, $n_{j}$, are then compared to each other. The remainder $n_{i}-n_{j}$ is used as a feature in the classification process, and this observation is labeled as “non-migraine day.” What is noticeable is that, in the latter cases, the difference between remainders $n_{i}-n_{j}$ and $n_{j}-n_{i}$ is only the sign. Therefore, in order to avoid over-fitting in the cross-validation process, these remainders were only calculated to one direction, and the sign *k* was randomly chosen. This, of course, causes contingency in the output matrix and therefore in the classification process. For this reason, classification results were calculated several times using every time different outputs of Algorithm 1. Finally, Algorithm 1 returns a night comparison feature matrix and the labels related to them.

Algorithm 1 clearly increases the number of observations. For example, suppose, in the collected data set, the number of nights before a day with migraine was *a*, and the number of nights before a day without migraine was *b*. In this case, by considering one night as one observation would mean that the whole data set would contain a+b observations. However, via Algorithm 1, and therefore considering the difference between nights as an observation, the data set contains a·b+(b·(b−1))/2 observations. Obviously, a·b+(b·(b−1))/2>>a+b. Therefore, Algorithm 1 increases the number of observations enabling the use of cross-validation in the classification process. In Table 2, it is shown study-subject-wise how many nights the data set contains and how many observations are generated from these nights using the approach presented in Algorithm 1 and Figure 1.

**Algorithm 1:** Algorithm to calculate night comparison features. Feature vectors $n_{i}$ are extracted from nights before a non-migraine day and vectors $m_{j}$ from nights before a migraine day.**input**: Feature vectors $F_{orig}=\left\{n_{1},\ldots ,n_{a},m_{1},\ldots ,m_{b}\right\}$
**output**: Night comparison feature matrix *F*, labels *L*
counter = 1; 
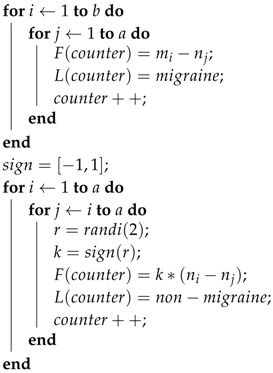

**return** feature matrix *F*, labels *L*;

Altogether, 110 features $f_{k}$ were extracted from the data of each night $n_{i}$ and based on these the final feature matrix was calculated using Algorithm 1. Features $f_{k}$ were calculated from raw signals, except acceleration features, which were extracted from magnitude acceleration signals. Features mostly include time domain features, such as standard deviation, mean, minimum, maximum, and median, which are commonly used to recognize human activities. These were calculated from each signal except heart rate variability measurements, the features extracted from which were those suggested in [23]. Note that the minimum was not calculated from galvanic skin response signals, as it was noted that it was always zero. Moreover, sleep time was divided into four equal-size parts, and differences in statistical values between the first and last parts were calculated and used as features. The first and last parts were considered the most interesting, as the period of time between the last part and the migraine attack was the shortest and the period between the first and the migraine was the longest, so differences between these parts were expected. All extracted features are listed in Table 3.

## 4. Early Recognition of Migraine Attacks Using Biosignals

In this study, two different classifiers were compared: quadratic discriminant analysis (QDA) and linear discriminant analysis (LDA). LDA is used to find a linear combination of features that separate the classes best. The resulting combination may be employed as a linear classifier. QDA is a similar method but uses quadratic surfaces to separate classes [24]. To avoid over-fitting in the feature selection phase, noise was injected into the data using the method presented in [25], and the most descriptive features for each model were then selected using a sequential forward selection (SFS) method [26].

**Figure 2:**
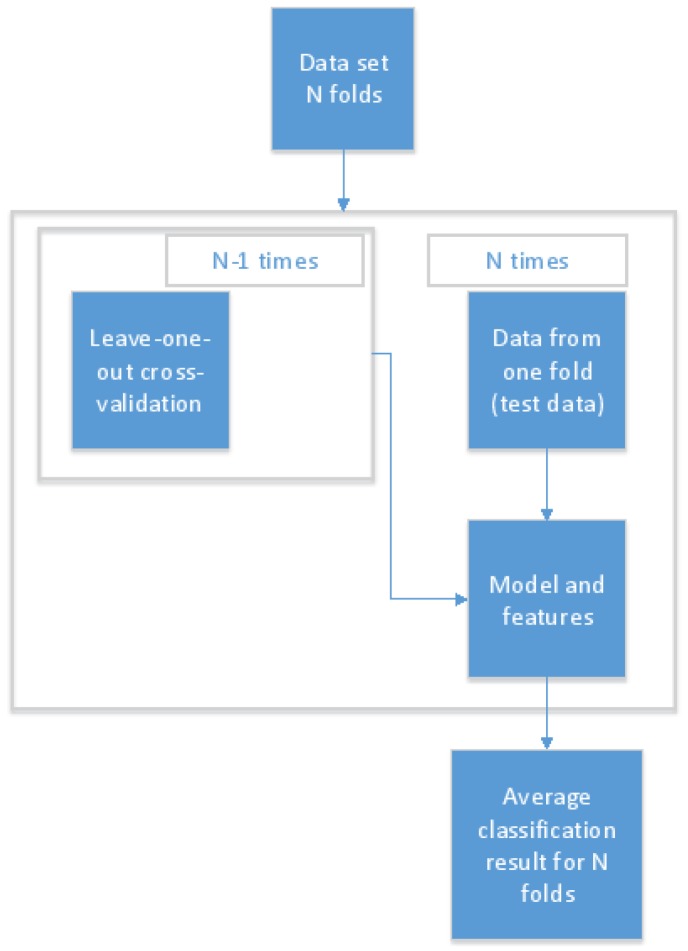
The model training and recognition process protocol [27].

To train reliable early detection models, the data set was divided into three sets: training, validation, and testing data. When personal models were built, the imbalanced structure of the data set was taken into account, and different rules were applied when selecting data points for the test set in the cases of nights before a day with or without a migraine. To avoid over-fitting, testing and training sets did not include samples calculated from the same night $m_{i}$ before a day with a migraine. Instead, all feature vectors that were calculated from $m_{i}$ using Algorithm 1 were selected simultaneously for the test set. For every test subject, two nights before a day with a migraine attack were randomly selected, after which Algorithm 1 was applied to these before adding them all to the test set. Therefore, the same nights before a day with a migraine attack were not used in the train and test data set. In the case of nights before a day without a migraine, the test set was created by randomly selecting 20% of the observations where nights before a day without a migraine were compared with each other. The rest of the data (training and validation) were divided into five parts, four parts for training and one for validation, and these were used to select the most descriptive features to build a reliable model. In the case of user-independent models, the over-fitting was easily avoided by using leave-one-out cross-validation: in turn, one person’s data were used as independent testing data and data from the other six study subjects were used for validation and training. The protocol used in the recognition process is shown in Figure 2 [27].

Due to the increase in data points using Algorithm 1 and random selection, samples calculated from a single non-migraine night can have from 0 to *a* observations in the test data set. Thus, the final classification was calculated by selecting all estimated class labels concerning night *x*, and the mode of these class labels was considered as the final classification result for the night *x*. Moreover, in each case, the average recognition accuracy was calculated in a similar way. Because classes are slightly imbalanced, balanced accuracies were calculated instead of normal accuracies. The balanced accuracies were obtained by calculating a true positive rate of both classes and by calculating the average of these.

## 5. Results

The comparison of the recognition results using personal and user-independent approaches are shown in Table 4. As Algorithm 1 has a random element, the classification results are not static. Therefore, for each tested setting, the results were calculated 20 times. For each run, a new feature matrix was generated using Algorithm 1. The standard deviation between these runs is shown in the results. It must be noted that they are not directly comparable with each other, while in the user-independent case all data from a single person at one time was used as testing data, unlike in the personal case where only a portion of single person data could be used.

The first observation that can be made from the results shown in Table 4 is that the balanced accuracies using user-independent models are much lower than the ones obtained using personal models. Moreover, when balanced accuracies of personal models are compared, it can be noted that QDA produces better results than LDA. In addition, standard deviation values show that balanced accuracies are highly dependent on which samples are selected for the train and test set.

The results using personal models and QDA classifiers are shown in more detail in Table 5. The sensitivity and specificity values of Table 5 show that, in the cases of Study Subjects 2 and 5, models do not work at all. In fact, they classify almost all instances into one class. Moreover, all the classification models are slightly biased toward correctly recognizing non-migraine days, as sensitivity values are higher than specificity values.

## 6. Discussion

The results of Table 4 show that early symptoms are highly personal because it was noted that user-independent recognition models cannot detect migraine attacks. In fact, the balanced recognition accuracies using user-independent models were below 50%. This came as no surprise as there are several different types of migraine attacks, and in this study only data from seven study subjects were used. Therefore, the user-independent results shown in this study are preliminary. To obtain better knowledge about how well a user-independent model can detect migraine attacks beforehand, data should be collected from much greater user groups or the study should concentrate only on one type of migraine attack. Moreover, it should be studied if there is variation in sensor readings between different devices. If each study subject wore a different sensor, differences in sensor readings would partly explain why user-independent models fail.

While a small data set is an issue for user-independent models, it is not that great a problem for personal models, as in such a case the problem is much more restricted. A personal model only needs to be able to detect only one type of migraine attack instead of all of them. When results using personal models are studied, it can be seen that recognition accuracy using QDA classifiers is promising and performs much better than LDA (84.1% vs. 70.2%). Therefore, is can be noted that the problem of detecting migraine attacks beforehand is more quadratic than linear. However, standard deviation values show that detection accuracies are highly dependent on which observations are selected in the train and test sets. Moreover, the subject-wise study of balanced accuracies shows that there is a great deal of variation between study subjects. In fact, the lowest balanced accuracy is 60.4% (Study Subject 2), while the highest balance accuracy is 95.2% (Study Subjects 3 & 6). Moreover, sensitivity and specificity values presented in Table 5 show that, in the case of Study Subject 2, the personal model does not work at all and classifies almost all observations into one class. The situation is almost the same with Study Subject 5.

The subject-wise study (see Table 4) shows that recognition rates can be divided into two groups: one for subjects (Subjects 1, 3, 4, 6, and 7) whose migraine attacks can be reliably detected beforehand using the methods used in this study, and the other for subjects (Subjects 2 and 5) whose migraine attacks cannot be detected accurately. Moreover, according to Table 5, personal models are biased towards recognizing non-migraine days with higher accuracy that migraine days, as sensitivity values are higher than specificity values. To understand why early detection is not accurate with all study subjects, it should be studied what is common with subjects and data collected from those whose migraine attacks can be detected beforehand and those whose attacks cannot be detected. These differences include, for instance, migraine types. It is possible that some types of migraine attacks are more difficult to recognize than others; therefore, more personal data should be collected from these study subjects to be able to recognize migraine attacks beforehand.

It is likely that the differences between study subjects are not the only reason for the large variance in balanced recognition rates. Environment can also cause differences in the data set, as environmental factors, such as an untypical smell, can trigger a migraine attack. Obviously, such an attack cannot be predicted beforehand. In addition, it should be studied if some of the false positive results are due to preventive medication. Moreover, the most feasible explanation for low recognition rates with some study subjects is the small data set. In fact, the high standard deviation values show that recognition accuracies are strongly dependent on which samples are selected in the train and test sets. These types of problems are typical when the used data set is small. Moreover, according to Table 2, Subjects 2 and 5, who had the lowest recognition rates, had the lowest number of migraine attacks during the data gathering session (five and six, respectively). However, this data cannot be fully used to train the early detection model, as such data need to be divided into training and testing data. It is quite understandable that, using so few samples, it is not possible to train a general and reliable early detection model. In the cases of Subjects 2 and 5, sensitivity and specificity values presented in Table 5 clearly indicate that the data set does not contain enough information to build a reliable classification model, as the model seems to classify almost all observations into one class.

One more problem is that it is possible that all data points were not correctly labeled. This is especially problematic when the data set is a non-comprehensive small data set, like in this study. The reason for false labels is that labeling was based on study subjects’ diaries where they described their symptoms and feelings using their own words. However, part of the future work is to improve the diary to get a better understanding about migraine attacks and the reasons for them. Now, at times, these descriptions were not unambiguous. For instance, study subjects reported that they had had a headache, but they did not always know if it was caused by a migraine or by something else. Therefore, labeling was subjective and not based on facts. While all of these challenges are, in one way or another, related to an available data set that is too small, it is also possible that the low recognition rates of some study subjects are due to the fact that the methods used in this study were not sophisticated enough to detect migraine symptoms from all study subjects. Therefore, the whole model training chain (pre-processing, feature extraction, feature selection, and modeling) needs to be studied and improved further if possible.

When the results and methods of our article are compared to the ones by Pagán et al. (e.g., [17,18], it can be noted that our data set is larger (two study subjects vs. seven study subjects) but also the approaches used in these articles are very different. Our study uses only a wrist-worn sensor and Pagán et al. use two devices (a finger-held SpO2 device and ECG patches attached), which is not an optimal solution for 24/7 monitoring. Moreover, our aim is to predict migraine days from the sleep time data, while Pagán et al. aim to predict the exact timing for a migraine attack. In fact, these two approaches can be considered as complementary: our approach could be used to predict days with a high risk of migraine attack, and during those days the solution suggested by Pagán et al. could be used to predict the exact timing of a migraine attack.

## 7. Conclusions and Future Work

Early detection of migraine attacks was studied based on sleep time data collected using wearable sensors. The data for the study were collected from seven study subjects, and on average there was 27 days’ worth of data from each subject. Moreover, the number of observations was increased using a novel approach where data size was increased by comparing differences between nights before a migraine day and nights before a non-migraine day. According to our preliminary results, the migraine attacks cannot be detected reliably beforehand using user-independent models. However, the used data set was most likely not comprehensive enough to build reliable user-independent models due to a limited number of study subjects. On the other hand, the small number of study subjects is not an issue when recognition is based on personal models instead of user-independent models. In fact, the results achieved using personal models indicate that early detection of migraine attacks is possible. When a personal early detection model based on QDA classifiers was used, the average balanced accuracy was over 84%. However, there is a great deal of variation between study subjects. In fact, when results are studied study-subject-wise, it can be seen that balance accuracy varies between 60.4% and 95.2%.

There are several possible reasons for high variation in recognition results between study subjects, and part of our future work is to determine these reasons. It is possible that some migraine types are more difficult to predict than others. Moreover, labeling of the data was challenging, as it was based on subjects’ diaries where they described their symptoms and feelings using their own words, and these description were not always unambiguous. Therefore, it is possible that not all samples are correctly labeled. Most importantly, more data should be collected to obtain a better understanding about the accuracy of user-independent models: here, there was data only from seven subjects, which does not cover all migraine types. Moreover, with a larger data set, it would be possible to experiment the accuracy of personal models with all migraine types. If the findings of this article can be confirmed in a larger population, it may potentially contribute to early diagnoses of migraine attacks.

## Figures and Tables

**Table 1:** Study subjects and their characteristics.

Study Subject	Age	Gender	BMI	Aura Symptoms	Type of Medication
1	30	male	21.7	yes	preventive
2	60	female	22.0	no	acute
3	32	female	39.1	no	preventive
4	47	female	22.4	no	acute
5	46	female	23.7	no	acute
6	47	male	23.6	yes	acute
7	48	female	29.0	no	acute

**Table 2:** Characteristics of the data set. The subject-wise number of available observations after the amount of data was increased using Algorithm 1.

Study Subject	Trial Duration (Days)	Migraine Days	Number of Observations
1	29	17	270
2	32	5	455
3	24	7	223
4	25	8	248
5	27	6	310
6	28	10	255
7	35	14	504
Total	200	67	2265

**Table 3:** Features used in this study. acc = accelerometer; bvp = blood volume pulse; temp = temperature; eda = electrodermal activity; hr = heart rate; hrv = heart rate variability.

Feature	Signal	Number of Features
Standard deviation	acc, bvp, temp, hr, eda, hrv	6
Mean	acc, bvp, temp, hr, eda, hrv	6
Minimum	acc, bvp, temp, hr	4
Maximum	acc, bvp, temp, hr, eda	5
Median	acc, bvp, temp, hr, eda	5
5th percentile	acc, bvp, temp, hr, eda	5
25th percentile	acc, bvp, temp, hr, eda	5
75th percentile	acc, bvp, temp, hr, eda	5
95th percentile	acc, bvp, temp, hr, eda	5
Comparing first and last hours of sleep: standard deviation	acc, bvp, temp, hr, eda	5
Comparing first and last hours of sleep: mean	acc, bvp, temp, hr, eda	5
Comparing first and last hours of sleep: maximum	acc, bvp, temp, hr, eda	5
Comparing first and last hours of sleep: minimum	acc, bvp, temp, hr, eda	4
Comparing first and last hours of sleep: median	acc, bvp, temp, hr, eda	5
Comparing first and last hours of sleep: 5th percentile	acc, bvp, temp, hr, eda	5
Comparing first and last hours of sleep: 25th percentile	acc, bvp, temp, hr, eda	5
Comparing first and last hours of sleep: 75th percentile	acc, bvp, temp, hr, eda	5
Comparing first and last hours of sleep: 95th percentile	acc, bvp, temp, hr, eda	5
Correlation between signals	acc, bvp, temp, hr, eda	14
Root mean square of time difference of adjacent heart beats	hrv	1
Mean of time difference of adjacent heart beats	hrv	1
Standard deviation of time difference of adjacent heart beats	hrv	1
Number of measured heart beats	hrv	1
The number of pairs of adjacent heart beats whose difference is more than 50 ms	hrv	1
Total power	hrv	1

**Table 4:** Recognition rates study-subject-wise using personal and user-independent models.

Study Subject	Personal Model (QDA)	User-Independent Model (QDA)	Personal Model (LDA)	User-Independent Model (LDA)
1	**91.2% (8.1)**	52.6% (2.3)	75.7% (10.4)	52.8% (3.1)
2	60.4% (13.5)	48.0% (0.6)	**62.0% (7.4)**	52.5% (4.7)
3	**95.2% (4.7)**	47.9% (5.5)	70.3% (7.4)	43.6% (5.5)
4	**94.9% (6.9)**	48.5% (5.3)	70.8% (12.5)	41.2% (5.4)
5	**69.6% (15.1)**	36.0% (6.6)	69.1% (9.0)	49.1% (2.8)
6	**95.2% (5.0)**	52.1% (6.2)	70.3% (8.7)	55.6% (6.6)
7	**82.0% (12.6)**	49.9% (2.6)	74.4% (7.6)	47.1% (4.7)
Mean	**84.1% (15.3)**	47.4% (7.5)	70.2% (9.8)	49.1% (7.7)

**Table 5:** Subject-wise accuracy, sensitivity, and specificity of personal recognition models using a quadratic discriminant analysis (QDA) classifier.

Study Subject	Accuracy	Sensitivity	Specificity
1	91.2% (8.1)	99.6% (2.0)	90.0% (20.5)
2	60.4% (13.5)	98.1% (2.4)	30.0% (34.0)
3	95.2% (4.7)	100.0% (0.0)	85.0% (38.9)
4	94.9% (6.9)	100.0% (0.0)	95.0% (15.4)
5	69.6% (15.1)	100.0% (0.0)	42.5% (33.5)
6	95.2% (5.0)	99.5% (2.2)	85.0% (28.6)
7	82.0% (12.6)	97.8% (3.6)	73.5% (25.7)
